# Analysis of the Factors Influencing Veterinary Food Inspectors in Poland

**DOI:** 10.3390/ani10050884

**Published:** 2020-05-19

**Authors:** Joanna Wojtacka, Beata Wysok, Joanna Szteyn

**Affiliations:** Department of Veterinary Public Health, Faculty of Veterinary Medicine, University of Warmia and Mazury in Olsztyn, Oczapowskiego 14, 10-917 Olsztyn, Poland; joanna.wojtacka@uwm.edu.pl (J.W.); szteyn@uwm.edu.pl (J.S.)

**Keywords:** inspection, hazards, crisis, official veterinarian, approved veterinarian, slaughterhouse

## Abstract

**Simple Summary:**

Veterinary public health is involved in various fields related to food animals, i.e., their welfare and health condition, food safety and quality. Proper management of animal health status, in slaughter and then monitoring procedures through the whole production chain, are incorporated in the work of Veterinary Food Inspectors. These inspectors are also involved in assessing epidemiological situation, taking decisions in crisis and supervising decision-making process in the slaughterhouse. Responsible for both animal and human health, Veterinary Food Inspectors struggle with moral and ethical dilemmas, conflicts with food industry workers and employment shortages. There is also growing demand for continuing education and training, in order to adjust veterinary service to dynamically changing food industry and to follow modern animal production. More attention must be paid to training in food hygiene subjects, given Veterinary Food Inspectors must have excellent competencies in this field, in order to guarantee excellent last contact with food animals.

**Abstract:**

The evaluation of the quality of Veterinary Inspection in Poland has received much attention in the past few years. Veterinarians working as Food Inspectors face numerous and newly arising problems in the protection of animal health status, providing surveillance information on the occurrence of diseases, and carrying out risk analyses of the hazards related to food of animal origin. From the 130 active veterinarians attending the post graduate courses in “Hygiene of slaughter animals, meat and animal origin products” in Poland, 119 took part in the survey. The questionnaire consisted of 15 questions that delivered information on demographic features of the respondents, and various aspects of the course of their work: (a) Motivation to undertake work in food safety sector, (b) overall job satisfaction, (c) crucial negative factors and (d) occupational hazards. Participants were mainly under 40 years of age. They were Veterinary Food Inspectors working as Official and Approved Veterinarians. Permanent position and economic reasons were their main motivation in the food safety sector. They indicated problems related to insufficient training in ante and post mortem examination, work with legal acts and risk analysis. They also declared a lack of preparation in coping with crisis situations. One third of the respondents declared their health and lives were endangered, while fulfilling professional duties and pointed at different sources of hazards. The overall evaluation of the work in food safety sector was rated good and satisfactory.

## 1. Introduction

According to national law, Veterinary Inspection (VI) in Poland carries out tasks in the field of animal health protection and safety of animal origin products to ensure public health [1]. In each country, the official Veterinary Inspection is organized on the basis of its specific tasks and the ‘history’ of national veterinary medicine [2]. The Polish system of food safety supervision is different from other UE countries, i.e., neighboring Lithuania [3], in terms of the structural organization and subject of supervision. In Poland the Chief Veterinary Officer (CVO) is appointed by the Prime Minister. Since 1999, the CVO has played the role of the main veterinary administration authority as a part of the Ministry of Agriculture and Rural Development. The inspection bodies are represented by Provincial, District and Border Veterinary Officers, served by their Offices, and working on the basis of a vertical chain of command. In the food safety sector, the activities and responsibilities of VI in Poland are focused on the procedures and supervision over slaughter animals, meat and other animal origin products, i.e., milk, eggs, honey, etc. according to the integrated ”from stable to table” approach. The safety of food of plant origin is supervised by other governmental bodies, i.e., Inspectorates for Plant Health and Seed Inspection or the Agricultural and Food Quality Inspection. There is also Sanitary Inspection that reports to the Minister of Health and supervises production, turnover and trade related to all food products. There have been some attempts in Poland to create consolidated inspection, mainly to eliminate jurisdictional collisions between the recently operating inspections and to eliminate issues, resulting from the double subordination of the VI at the country level [4]. However, works on the project of common National Food Safety Inspection has been currently suspended.

There are 5600 people, including more than 3000 veterinarians, employed in VI in Poland, 200 of which work in official veterinary laboratories. Additionally, more than 5700 veterinarians are appointed by the District Veterinary Officers to the tasks related to eradication of infectious diseases and examination of slaughter animals and meat [5]. According to legal definitions [6], veterinarians working in food safety sector are though divided into two groups. There are Official Veterinarians (OVs) working in the Inspectorates on permanent positions with fixed wage paid from the state budget and Approved Veterinarians (AVs) appointed to specific activities for limited periods, and remunerated on the basis of an officially fixed rate, i.e., per animal examined.

For the past several years VI in Poland has struggled with numerous problems starting from the years 2006/2007 when first H5N1 cases and outbreaks were noted [7] until today, with constantly spreading African Swine Fever (ASF) and new outbreaks of Avian Influenza (AI) at the beginning of 2020. All above have pulled other burning problems concerning service of VI into the daylight. The report published by EU Health and Consumers Directorate [8] already indicated relatively low level of remuneration, which had led to a turnover of Polish veterinarians working in VI who moved to higher paid jobs outside once they had been trained. Currently, it is estimated that there are approximately 300 vacancies for veterinarians in VI, that is not only an institution responsible for human and animal health and safety of all animal products, but also for the whole agricultural market and the Polish food export derived from it. Apart from the externally-born obstacles arising from: (a) Epidemiological situation, (b) economic (salary) and (c) employment deficiencies. There are also other internally-borne factors that deeply influence the perception of VI as a workplace of veterinarians. Stress at work, conflicts and burnout have been reported in vets practicing clinical veterinary medicine [9,10,11,12,13,14], but there is no data on their input and scale in VI, including veterinary food safety inspection.

The aim of the study was to recognize the motivation of veterinarians in undertaking work as Veterinary Food Inspectors (VFI) and characterize the impact of crucial negative factors (deficiencies in knowledge or practice in some thematic area, work related conflict) and occupational hazards (crisis situation, burnout, life-threatening situations) they can encounter in the course of work on overall job satisfaction.

## 2. Materials and Methods

### 2.1. Questionnaire Survey

A survey of active veterinarians, practicing in Poland for at least 2 years, was conducted at the end of 2019. All respondents have been enrolled in four-semester post-graduate courses in “Hygiene of slaughter animals, meat and animal origin products” available for active vets. The courses are held in Warsaw (Warsaw University of Life Sciences), Olsztyn (University of Warmia and Mazury in Olsztyn), Puławy (National Veterinary Research Institute) and Wrocław (Wrocław University of Environmental and Life Sciences). The respondents filled in the survey anonymously and voluntarily during one of the course meetings, included in the curriculum. All 130 surveys were distributed among the whole group and analyzed. The survey consisted of 15 questions. The first three questions served as supporting information concerning demographic features describing our respondents, i.e., the type of work performed, gender and age group. The remaining questions concerned various aspects of the course of work in the sector of food safety. The questionnaire survey asked about: (a) Motivation to undertake work in food safety sector, (b) overall job satisfaction, (c) crucial negative factors, i.e., deficiencies in knowledge or practice and work-related conflicts, (d) occupational hazards, i.e., crisis situations, life-threatening situations and burnout. They were close-ended questions, however, the “other” field provided the opportunity to include their own statement and answer the 5 questions. The respondents could also select more than one answer in these 5 cases. At the survey distribution stage, no attempt was made to structure the participating veterinarians as the study was designed to use an opportunistic sampling configuration [15].

The completed questionnaires were coded numerically, arranged into data sets, and subjected to further analyses.

### 2.2. Statistical Analysis

The statistical analyses were conducted using Statistica StatSoft. In the case of nominal values, the differences between the groups (OVs and AVs) were analyzed with Pearson’s Chi-square test, which was applied to the data, retrieved from the answers regarding place of employment, motivaon to change the workplace, crisis situation in the workplace, and risk for life and health. In case of multiple pairwise comparisons, Bonferroni correction was implemented, which applied to the responses to the questions regarding the most difficult topics, motivation to perform work in food safety sector, experience of conflict in the workplace and factors that could influence work satisfaction. For ordinal values, the Mann-Whitney *U*-test was used as data were non-normally distributed, i.e., in terms of overall job satisfaction, the frequency of conflict in the workplace and burnout. Statistical significance was defined as *p* < 0.05. 

## 3. Results

### 3.1. Demographic Features

Basic numerical data is given in Table 1. One hundred and nineteen individuals responded to the survey (91.5% response rate). The respondents came from all over Poland. The majority of the veterinarians surveyed were females. More than a half of the participants of the course was 25 to 35 years old. The majority, which was 105 respondents (88.2%) consisted of the vets working as VFIs. There were 65 female and 40 male VFIs that accounts for 54.6%, and 33.6% of all respondents, respectively. The group of VFIs was also divided into OVs (*n* = 53), and AVs (*n* = 52) that accounted for 50.5% and 49.5% of all VFIs, respectively. There were 65 women (*n* = 65) employed as VFIs compared to 40 men, that accounted for 62%, and 38%, respectively in this group. Women were statistically more often represented OVs than AVs (*p* < 0.05) than men. A total of 31 (26.1%) respondents, working as VFIs, work afterhours in veterinary practice where they are both owners and employees. Veterinary School was the workplace of 4 (2.5%) responding vets. Also 4 (2.5%) veterinarians declared that they work in companies producing feed and involved in animal breeding. There was also one VFI in this group. Only 1 woman (0.6%) was employed in a VI laboratory. 

### 3.2. Motivation of Veterinarians to Undertake Work in Food Safety Sector

When asked why they perform work related to food safety, the respondents could tick more than one answer. Consequently, 148 different answers were obtained. The highest number of the answers (*n* = 43, 29%) given by the VFIs indicates that respondents were financially driven, ”this job is well-paid”. AVs (*n* = 38) pointed at this answer statistically significantly (*p* < 0.05) more often than OVs (*n* = 5). Statistically significant differences (*p* < 0.05) were also noted between two groups of VFIs, in terms of other three answers, i.e., indicating that they always wanted to work in the field of food safety. There were 14 AVs and 6 OVs, who marked this option. The other answer concerned the possibility to have permanent position at work and there were 31 OVs, and 5 AVs who choose that answer. Other answers indicating ”no other possibilities for employment at the place of residence”, own curiosity, variability of this work, plans to change for work in food safety sector, this work is additional occupation, and service for the public health were marked by 9 OVs and 2 AVs, that accounted for 7.4% of all the answers given to this question. Statistically significant differences (*p* < 0.05) were also noted between female and male VFIs in terms of financial and permanent position motivation to perform their work. Within this group male respondents (*n* = 24) were more often financially driven than females (*n* = 19). On the contrary fixed position was more frequent motivator for female VFIs (*n* = 29) than males (*n* = 7).

### 3.3. Overall Job Satisfaction

There were three questions regarding job satisfaction. In the first one we asked what grade the respondents would give to their workplace. The majority (54.6%) decided for grade, “good”. For 43.5% respondents it was “satisfactory”, 6.7% indicated “very good”, and 4.2% ticked grade “unsatisfactory”. In the whole group analyzed VFIs (*n* = 105) assessed their work mainly as “good” (*n* = 55, 52.4%), and “satisfactory” (*n* = 42, 40%). Five FVIs (4.8%) pointed at grade, “very good”, and 3 vets (2.9%) pointed at “unsatisfactory”. There were no statistically significant differences (*p* > 0.05) noted between OVs and AVs, in terms of their perception of overall job satisfaction.

The second question was about significant factors, excluding money that would influence work satisfaction of veterinarians working in food safety sector. The respondents could tick more than one answer. Consequently, 295 different answers were obtained. Simplification of administration procedures received the highest number of votes (*n* = 83) that accounted for 28.1% of all the answers given to these questions. Training for farmers and employees of food industry in their cooperation with VI was selected 67 times (22.7%). ”Higher number of additional training for employees of VI” (*n* = 54) and “increased number of posts” (*n* = 50) accounted for 18.3%, and 16.9% for each answer, respectively. Statistically significant difference (*p* < 0.05) was observed between the number of the latter answer given by the AVs and OVs. OVs (*n* = 35) statistically significantly more often pointed at the need to increase the number of posts than AVs (*n* = 11) that shows 33.3% to 10.1% ratio in the group of VFIs. Thirty three answers (11.2%) indicated “including the latest European Food Safety Authority (EFSA) guidelines in training topics”. Other answers (*n* = 8) written by the respondents themselves accounted for 2.7%. They were as follows: Excluding VI from the dependence on Ministry of Agriculture and Rural Development, decent working conditions, clear distinction of veterinarians from other employees of VI, system changes in VI, training in soft skills, i.e., conflict management, managing of work relationships. 

In the last question, the respondents were asked whether they consider a change in workplace to be one that would not be related to food safety sector. The positive (*n* = 62, 52.1%) and negative (*n* = 57, 47.9%) answers to this question were distributed equally. There was also no statistically significant difference (*p* > 0.05) in terms of their plans for their professional future in females and males both for all respondents and the group of VFIs. The number of 22 and 13 women working respectively as OVs and AVs think about the change, and 22 and 8 do not plan to change the job. The number of 4, and 14 men working, respectively as OVs and AVs consider changing their workplace, 5 and 17 do not think about any change. 

Similar to the distribution of positive and negative answers to the above question, there were no statistically significant differences (*p* > 0.05) between VFIs employed as OVs and AVs. There were 26 OVs and 27 AVs declaring their will to change the present job in the Inspectorate and appointed position, and 27, and 25 VFIs giving negative answer, respectively.

### 3.4. Crucial Negative Factors

**Figure 1:**
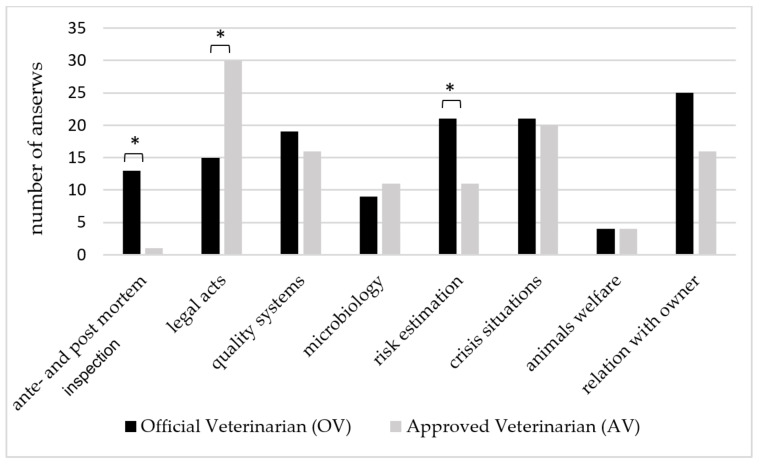
The topics that were declared as the most difficult for Veterinary Food Inspectors (VFIs). * Asterisks indicate statistically significant differences (*p* < 0.05) between OVs and AVs.

There were three questions concerning negative factors related to the workplace of our respondents. First, we asked about the topics that are most difficult for the veterinarians taking part in the survey. The respondents could tick more than one answer. Consequently, 263 different answers were obtained. Top three answers have covered: Ability to work with legal acts and knowledge of regulations (*n* = 54), managing crisis situations (*n* = 48), and relations and contact with animal owners/employees of the food industry (*n* = 46) that accounts for 20.5%, 18.2%, 17.5% of all the answers received to this question. However, statistically significant differences (*p* < 0.05) were noted in the group of VFIs in terms of practical ante and post-mortem examination, which was not problematic for AVs (only 1 declared difficulties in this matter). On the contrary, 13 OVs declared it was difficult for them. The opposite tendency was noted while the ability to work with legal acts was analyzed. Difficulties in this area were declared by 15 OVs and 30 AVs, which also made statistically significant difference (*p* < 0.05) between the groups. The latter answer that produced statistically significant difference (*p* < 0.05) concerned risk analysis of food hazards that was problematic for 21 OVs and 11 AVs (Figure 1). Other answers concerned difficulties with the topic related to safety and quality assurance systems in the food industry that were reported 37 times (14.1%) and food microbiology (*n* = 20, 7.6%), and welfare of slaughter animals (*n* = 8, 3%).

Next, our respondents were asked whether they experience conflict in their workplace. The number of 110 respondents (including 94 VFIs) responded positively. Among VFIs the conflict is encountered frequently by 17 respondents (16.2%), moderately often by 40 (38.1%) respondents, rarely by 37 (35.2%) respondents. Eleven (10.5%) VFIs do not experience conflict at work. However, no statistically significant difference (*p* > 0.05) was noted between OVs and AVs.

**Figure 2:**
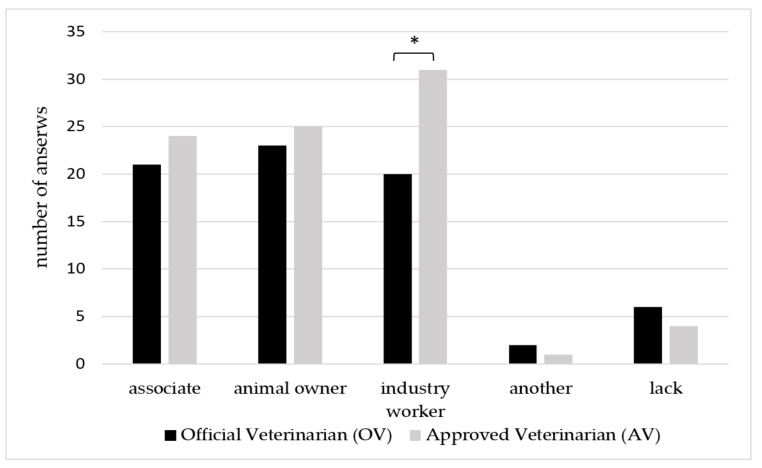
The opposing party of the conflict Veterinary Food Inspectors (VFIs) experience in their work. * Asterisks indicate statistically significant differences (*p* < 0.05) between OVs and AVs.

When veterinarians were asked about the opposing party, they could tick more than one answer. Consequently, 161 answers were obtained. The respondents indicated animal owners/farmers (*n* = 55), co-workers (*n* = 47) and employees in food industry (*n* = 53), that accounts for 34.4%, 31.2% and 31.2% of all the answers received for this question. Six (3.7%) vets indicated other opponents, i.e., hunters, local government, and superior authorities of VI. Statistically significant differences (*p* < 0.05) were noted in the group of VFIs, in terms of employees in food industry that were indicated to be the opposing party in conflict situations experienced by AVs (*n* = 31), and OVs (20) that accounts for 29.5% and 19% of all VFIs (Figure 2).

### 3.5. Occupational Hazards

There were two questions regarding the crisis situation in the survey. We asked whether the respondents had already encountered a crisis situation in their work. Generally, 48 vets (40.3%) responded positively. They were almost all VFIs (*n* = 45), 25 of whom were OVs and 20 were AVs. They reported such crisis situations as: Eradication of swine herds and epizootic investigation in the disease outbreaks due to ASF, eradication of other infectious diseases, i.e., Enzootic Bovine Leucosis (EBL), salmonellosis in laying hens, avian influenza, American foulbrood, suspicion of tuberculosis, trichinosis, fire in the food processing plant, fire in the chicken houses, fire of a livestock building, failure of slaughter process, illegal production and processing of meat products, construction disaster in a chicken house, collision of vehicles transporting animals, slaughter of sick and dying animals and flood. When asked whether they felt prepared to cope with such situations, the majority of VFIs (*n* = 63) responded negatively. Readiness to manage crisis in public health was declared by 42 VFIs surveyed. There was no statistically significant difference (*p* > 0.05) between OVs and AVs in terms of both previous experience in a crisis situation and preparation to cope with crisis situations. 

Next, two questions considered burnout, which was shortly described in the survey as exhaustion, lack of commitment, and lack of expectations. We asked whether the respondents felt burnout and whether working in food safety sector predisposed to this condition. Sixty seven respondents (56.3%) declared they do not suffer from burnout, 41 (34.4%) felt they were close to burnout, and 11 (9.2%) declared burnout. Generally, among the VFIs, 59 vets declared no burnout, 37 declared they were close to burnout, and only 9 felt burnout. There were statistically significant differences (*p* < 0.05) between AVs and OVs in the perception of burnout experience. The number of 34 AVs and 25 OVs did not experience burnout problem, condition close to burnout was declared by 16 AVs and 21 OVs, and burnout was experienced by 2 AVs and 7 OVs. 

**Figure 3:**
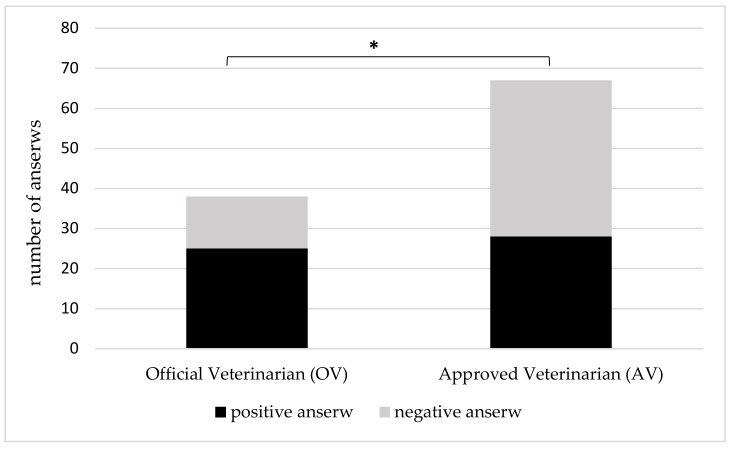
Differences in the Veterinary Food Inspectors (VFIs) experience of health and life threatening situations while fulfilling professional duties. * Asterisks indicate statistically significant differences (*p* < 0.05) between OVs and AVs.

Finally, our respondents were asked whether their lives and health had been at risk while fulfilling professional duties. Eighty three (69.7%) respondents replied they had not, and 36 (30.3%) replied that they had. The ratio of positive to negative answers to these questions among VFIs was 38 (36.2%) to 67 (63.8%), respectively, which was statistically significantly different (*p* < 0.05) (Figure 3). Our respondents (VFIs) enumerated numerous hazards of industrial, environmental, and professional origin that they had already experienced (Table 2). 

## 4. Discussion

From the turn of the 20th and 21st century, most countries have experienced resource reduction in their veterinary services. The effect of the policies at the source of these reductions has been severe, in many cases leading to an inability of veterinary services to conduct their disease prevention and control duties [16]. There are approximately 9000 vets working as VFIs in Poland, and more than a half of AVs is appointed for more than one activity. The data provided by the Federation of Veterinarians of Europe [17] shows that the avarage age of veterinarians is under 40, which is in line with the average age of our respondents and indicates that VFIs similarly to vets working in other fields of veterinary medicine are at the beggining of their careers. The above raise concerns about uncontrolled staff exchange in VI, becuase one of the most important factor associated with leaving a place of employment by veterinarians has been identified as a lack of support and menthorship [18] that is inevitable in younger teams. Moreover, formerly reported feminization of veterinary profession [19], is also visible in the VI in Poland. It applies mostly to the permanent position ensured by the employment as OV in the Inspectorate. This turns out to be one of the main causes to undertake work in VI by veterinarians. The majority of OVs are not motivated by goals like protection of public health or special interest in food hygiene and food safety problems that were declared by AVs. At this point, it should be underlined that AVs as well-paid and free practising group of VFIs in Poland may show different perception of their work in veterinary public health. The above clearly creates undesired division within the group of Polish VFIs. However, the diversity of political, economic and social situations which exist in and between countries dictates that no one model of organisational structure of Veterinary Services can be applied to all circumstances [20]. Taking these two advantages (permanent position and high remuneration) into account both groups, OVs and AVs, show a similar share of opinions on how they are satisfied with their work, with the majority evaluating their workplace as good and satisfactory. Both, OVs and AVs see administration procedures as too complicated. All of them also declare problems in a lack of proper training of farmers and employees of food industry in the cooperation with VI. However, these are mainly OVs that suffer from staff shortage in VI in Poland. This undesirable condition is alarming as VFIs, overwhelmed by multiple duties and laborious administration procedures, become detached from their real tasks. It may result in moral hazard with significant effects on their behaviour, which may even unintentionally become more cursory to the detriment of public health [21].

Our results also show that VFIs do not feel fully trained and prepared in all aspects of food hygiene. Their self-confidence and knowledge comes in time, based on professional practice, while most of the skills related to food safety should be included in ”day one competences” package of the properly prepared graduate. Although, it can be difficult to obtain because, i.e., meat inspection practices involve multiple checks. Additionally, the rapid increase in the average herd size has had consequences for the workload of many parties including meat inspectors [22]. Our results show that ante and post-mortem inspection was more challenging for OVs, who deal more with office and administration work, than for AVs who, in turn, find work with legal acts more difficult. New EU legislation that has recently come into force, updates the regulation of ”hygienic package”. However, it will not solve this problem in the forthcoming months. Moreover, both, OVs and AVs find difficulties in risk analysis and assessment that together with methodologies are at the heart of modern approaches to food safety. It is also VI that must adopt new approaches to decision-making and standard-setting if they are to be successful risk managers [23]. In our study, the OVs listed risk assessment as a topic causing difficulties more often than the AVs who work in the field or directly in the food producing plant or the slaughterhouse. This indicates that office work makes veterinarians separated from the circumstances arising on daily routine in food safety sector. Thus, all forms of veterinary education ought to conform to the current needs and take into account that, nowadays, one of the tasks most frequently requested of veterinary professionals is to guarantee the safety of food production for the consumer, by safeguarding human health without neglecting animal health and welfare [24].

Interestingly, over 40% of VFIs taking part in our study have experienced crisis situation in their work. They were both AVs who are most commonly at the place in times of crisis, and OVs, who have to manage and proceed with all issues regarding such situation. Nevertheless, much attention ought to be also drawn on the fact that the majority of VFIs surveyed do not feel prepared to cope with crisis situation. In the context of national health structures, these are the official Veterinary Services that must play the role of ”guarantor”, i.e., these Services must ensure that all problems related to the activities and fields of competence of veterinary medicine are managed effectively, in such a way as to uphold the rights and health care standards of all citizens [2]. This shows that much work must be completed for training VFIs in Poland because coping with crisis situation requires multidimensional approach. It consists of a special crisis division, refined crisis protocols, creation of training and simulation programs, developing the use of personal protective equipment and finally creation of quick response teams [25]. Such widespread actions also have international impact, given that proper training and functioning means veterinary inspection services allow all countries to meet trade obligations and access to the international markets by ensuring that food safety and animal health control are effective and trustworthy [26]. The above applies to routine work of VFIs and especially to crisis situations when the spread rate of hazards is significantly increased. Moreover, there is strong correlation between crisis, hazards and arising conflicts. According to our results, conflicts in VFI workplaces occur but their frequency is not related to the type of employment. However, AVs conflict more often with the employees in the food industry than the OVs that do not do field work on regular basis. Taking all the above deficiencies into account, it is probable that VFIs are prone to burnout that has already been described in veterinarians practicing with pets, farm animals and mixed practices all over the world [27]. It has also been reported [10] that burnout occurs in veterinarians of all specialties. Our study shows that the perception of burnout and experiencing it are different within one specialty group as OVs and AVs experience it differently. Undoubtedly, health and life risks that are incorporated in the work of veterinarian, contribute to burnout in this profession. Our respondents pointed numerous hazardous situations and factors that they encountered in their work. Generally, veterinarians are challenged by an imposing group of occupational hazards. However, among recognized and widely-reported exposure to ionizing radiation, injury (scratches, bites, kicks), allergy, biological agents and chemicals in veterinary practice [28], work-related and life-threatening hazards of VFIs have not been well-studied. In this context dramatic events that took place in the Netherlands in 2001 during the foot-and-mouth-disease crisis are worth mentioning. Angry farmers used violence against veterinary inspectors, taking some of them hostage and hanging dead animals, with the names of crisis managers on trees [25]. VFIs that took part in our survey also pointed to aggressive attacks from breeders, owners and controlled parties, classifying them into the group of health and life hazards. Thus, it is noteworthy that except from hazards that are related directly to the specifics of veterinary profession, i.e., contact with animals, biological material, etc., many of the hazards come from food business operators and their facilities or directly with the animal owners, breeders or people employed in the food industry, which was indicated by the VFIs in our survey. 

Despite clear inconsistency between the demands placed on veterinary services and the current level of funding and support they are receiving [16] Veterinary Services must demonstrate their ability to act [2]. Current work conditions of VFIs and crises related to ASF in Poland made veterinarians strike and demonstrate their inability to act, which is highly worrisome. If not filled, staff shortages may lead to deterioration of efficiency and efficacy of VFIs. According to Terrestrial Animal Health Code [29] human resources in Veterinary Services are subjected to evaluation. They must consist of an integral core of full-time civil service employees and this core should always include veterinarians complemented by administrative officials, veterinary para-professionals, part-time and private sector veterinarians and veterinary para-professionals. Following the above, in Poland both, OVs and AVs are subjected to legal disciplinary provisions. Except from unified legal requirements towards all VFIs in Poland there are also professional solidarity and the fact that most VFIs working in one region know each other personally. VFIs in Poland evaluate their workplace in a positive way and do not consider it the main source of conflict in their work, which is in line with assumption that veterinarians should be open to the plurality of moral beliefs and be willing to actively search for new ways to deal with the conflicting expectations [30]. This shows strong points of VFIs that stay integrated despite many obstacles arising from political and socio-economical sources. Our study shows some limitations, including the use of multiple choice answers and providing the opportunity for participants to include their own comments which resulted in various different responses. Our sample was not large, and it represents situation in Poland, which may not be transferrable to other countries. However, this is first study aimed at identifying the numerous problems of veterinarians working as Food Inspectors. We recommend further studies in other EU countries since all of them operate based on unified legal acts and requirements in terms of veterinary supervision of food safety. 

## 5. Conclusions

The VFIs in Poland are mainly young veterinarians that are motivated to work in VI by the possibility of gaining a permanent position (OVs) of employment and a high income (AVs). They find a balance between the advantages and disadvantages of their work, however, the tendency in deterioration of work conditions and small participation of experienced staff, working as VFIs, was noticed. Thorough under- and post-graduate training in the field of food safety, together with providing employment corresponding to the demand of public health, is the main policy to make VI grounded and efficient. 

## Figures and Tables

**Table 1:** Demographic features of veterinarians attending postgraduate courses in “Hygiene of slaughter animals, meat and animal origin products”.

Questions (Q) and Answers	*n* (Answers Received)	Percent
Q1. What is the type of your employment? (more than one answer was acceptable)		
Owner of veterinary practice	19	11.9%
Employee in veterinary practice	21	13.1%
Employee in Veterinary Inspectorate (Official Veterinarian)	53	33.1%
Employee in laboratory of Veterinary Inspection	1	0.6%
Veterinarian nominated for official activities (Approved Veterinarian)	57	35.7%
Employee in laboratory (other than Veterinary Inspection)	0	0%
Employee in Veterinary School	4	2.5%
Employee in the company involved in animal farming/breeding/turnover, pharmaceuticals, feed production	4	2.5%
Other	1	0.6%
Q2. What is your gender?		
Female	73	61.3%
Male	46	38.7%
Q3. Select your age.		
25–35	70	58.8%
36–45	36	30.3%
46–55	12	10.1%
56–65	1	0.8%
Over 65	0	0%

**Table 2:** Hazards encountered by Veterinary Food Inspectors (VFIs) while fulfilling their professional duties.

Hazadrs of Industrial Origin	Hazards of Environmental Origin	Hazards of Professional Origin
• Contact with sharp tools when working on the line (cuts when examining animals) • High concentrations of harmful gases • Gas leaks • Passing under the slaughter line • Fall of the slaughter line • Work at low temperature • Noise • Slippery floor • Carcasses falling from a hook at the examination point	• Threats during inspection • Intimidation • Vulgar and aggresive animal owners, petitioners and breeders • Verbal attacks • Elbowing with breeders • Aggressive attacks from the party subjected to control	• Work with biological material • Contact with wild animals • Occupational diseases, i.e., respiratory infections associated with work at poultry slaughter • Inadequate taming of the animal during examination, animal attacks • Sanitary slaughters • Lack of support staff during cattle examination
